# Changes in physical activity during transition to retirement: a cohort study

**DOI:** 10.1186/s12966-016-0375-9

**Published:** 2016-04-16

**Authors:** Sari Stenholm, Anna Pulakka, Ichiro Kawachi, Tuula Oksanen, Jaana I. Halonen, Ville Aalto, Mika Kivimäki, Jussi Vahtera

**Affiliations:** Department of Public Health, University of Turku, Turku, Finland; School of Health Science, University of Tampere, Tampere, Finland; National Institute for Health and Welfare, Helsinki, Finland; Department of Social & Behavioral Sciences, Harvard T.H. Chan School of Public Health, Boston, MA USA; Finnish Institute of Occupational Health, Helsinki, Finland; Department of Epidemiology and Public Health, University College London Medical School, London, UK; Clinicum, Faculty of Medicine, University of Helsinki, Helsinki, Finland; Turku University Hospital, Turku, Finland

**Keywords:** Physical activity, Retirement, Aging, Cohort Study

## Abstract

**Background:**

Retirement is a major life transition which may affect lifestyle. The aim of this study is to examine within-individual changes in physical activity during the transition from full-time work to retirement.

**Methods:**

The study population consisted of 9,488 Finnish public-sector employees who retired in 2000–2011 and who reported their leisure-time and commuting physical activity before and after retirement. On average, participants provided data at 3.6 (of the four) repeat examinations during 10 years before and 10 years after the retirement. Physical activity was self-reported and was expressed as weekly metabolic equivalent task (MET) hours. Generalized estimating equations were used to examine physical activity trajectories around retirement.

**Results:**

Among participants entering to statutory retirement physical activity first increased by 1.81 MET-hours (95 % confidence interval [CI] 1.20 to 2.42) during 4-year retirement transition, but then decreased by −1.80 MET hours (95 % CI −2.83 to −0.79) during the subsequent post-retirement period. Older retirement age, higher occupational status and fewer chronic diseases were associated with greater increase in physical activity during transition to statutory retirement.

**Conclusions:**

Statutory retirement appears to be associated with a temporary increase in physical activity. Future research should examine ways to maintain the increased activity level after retirement.

**Electronic supplementary material:**

The online version of this article (doi:10.1186/s12966-016-0375-9) contains supplementary material, which is available to authorized users.

## Background

A quarter of the population in most of the European countries will be aged 65 years or more by 2020. Currently large postwar baby-boomer generations are retiring from labor market into the “third age” [1]. The transition to retirement is considered as a major life event which may change people’s daily routines and affect health behaviors, including daily physical activity [2, 3]. Physical activity is one of the key components of active and healthy aging [4, 5]. However, it remains unclear how retirement shapes physical activity behaviors.

Some previous studies have reported that retirement is associated with an increase in leisure-time physical activity but also with a decline in overall physical activity [6–9]. To our knowledge, only one previous investigation, the French GAZEL study, has monitored physical activity levels repeatedly around the retirement transition [8], but the results might not necessarily be generalizable to other working populations because the large majority of GAZEL participants retired already at age 55 years with good pensions. A further limitation of the previous studies is imprecision due to the reliance on few repeat measurements, typically only one measurement before and one after retirement [7, 9, 10]. In addition, little is known about factors that may modify the effects of retirement on physical activity.

To address some of these limitations, we collected individual-level repeat data around retirement and examined physical activity trajectories among aging workers transitioning from full-time work to statutory retirement. In addition, we examined a range of pre-retirement factors, such as retirement age, sex, occupational status, life-style factors and chronic diseases, as potential modifiers of the retirement and physical activity association.

## Methods

### Study population

The data were from the Finnish Public Sector study, an ongoing prospective cohort study with identifiable questionnaire surveys. The eligible population of the original cohort included all employees who had been working for a minimum of six months in the target organizations, which included ten towns and six hospital districts, between 1991 and 2005 (*n* = 151,901) [11]. Nested survey cohorts included all those who were employed by the participating organizations at the time of surveys or had left the organizations after participating in an earlier survey, repeated by 4-year intervals. Surveys used in this study were collected in 2000-2002, 2004/2005, 2008/2009 and 2012/2013. Survey data for cohort members were successfully linked to employers’ records and comprehensive national health registers through unique personal identification codes, which are assigned to all citizens in Finland. The FPS study was approved by the Ethics Committee of the Hospital District of Helsinki and Uusimaa.

Of the cohort members, we identified persons who were at work and responded to at least one survey in 2000/2002, 2004/2005 or 2008/2009 (*n* = 81,587). Of those employees, 19,058 persons retired between 2000 and 2011. For this study we included persons who retired at statutory retirement age (statutory retirement, i.e., old age retirement), persons who entered to part-time retirement not due to health reasons (part-time retirement) and person who retired early on health grounds (from now on called as disability retirement). In case of entitlement to several pensions from different pension schemes at different times, the first one of awarded of above mentioned retirements types was selected.

For analytical purposes, the day of actual retirement was set as 0. From that point the duration to all previous and subsequent waves was calculated (-10, −6, −2, +2, +6, +10). In this study the pre-retirement period constitutes years −10 and −6, retirement transition years −2 and +2 and post-retirement period years +6 and +10. The difference between each wave is four years and years are averages across study population.

For this study, we included participants who had data on physical activity immediately before and after retirement (years −2 and +2) comprising a sample of 9,488 persons (80 % women). On average, participants provided physical activity data at 3.6 (range 2–4) of the possible four study waves.

### Assessment of retirement

Data on retirement were obtained from the Finnish Centre for Pensions, which coordinates all earnings-related pensions for permanent residents in Finland [12]. All gainful employment is insured in a pension plan and accrues a pension; thus the pension data with successful linkage were available for all participants. The start dates for any pension were obtained for all participants from 2000 through 2011, irrespective of the participants’ employment status or workplace at follow-up.

According to the public sector Employees’ Pension Act, the *statutory retirement* age was generally from 63 to 65 years until 2005 and 63 to 68 years from 2005 onwards, although some individuals had kept their earlier retirement age from the previous pension act in which pension ages in some occupations were below 63 years (e.g., 60 years for primary school teachers, 58 for practical nurses). *Part-time pension* may be granted to a person who is at least 60 years old and who is transitioning from full-time work to part-time work. A *disability pension* may be granted if, due to an illness or injury, the employee cannot continue working even after attempts of rehabilitation, re-education, or assistance. In Finland employees may apply for disability pension when more than 300 reimbursed sickness absence days have accumulated during two consecutive years on the basis of the condition causing work disability. Around 70–80 % of all disability pension applications have been accepted [13].

### Assessment of physical activity

Physical activity was assessed identically at each study wave. The respondents were asked to estimate their average weekly hours of leisure–time physical activity (including commuting) within the previous year in walking, brisk walking, jogging, and running, or their equivalent activities [14]. Each intensity grade had five response alternatives of which the class mid–points were used for the calculation of time spent in physical activity: no activity, less than 0.5 h (15 min used for calculation), ~1 h (45 min), 2–3 h (2.5 h), and ≥ 4 h/week (5 h). Cronbach’s alpha for the response categories was 0.68. The time spent on activity at each intensity level in hours per week was multiplied by the average energy expenditure of each activity, expressed in metabolic equivalent (MET). For example, walking, brisk walking, jogging and running corresponded to 3.5, 5, 8 and 11 METs, respectively [15]. In addition, the time spent (hours/week) in moderate intensity physical activity (walking, and brisk walking, or their equivalent activities) and vigorous physical activity (jogging, and running, or their equivalent activities) were calculated separately. Based on current physical activity recommendations the respondents were also classified as inactive if the weekly physical activity was less than 14 MET hours per week [16].

### Assessment of covariates

Sex and occupational title were obtained from the employers’ registers. Based on occupational titles, occupational status was categorized to upper-grade non-manual workers (e.g. teachers, physicians), lower-grade non-manual workers (e.g. registered nurses, technicians), and manual workers (e.g. cleaners, maintenance workers) [17], and was determined by the last occupation preceding retirement.

Life-style related factors, that is body mass index (BMI), smoking and alcohol use, were based on the last questionnaire prior to retirement (wave -1). BMI was calculated from self-reported weight and height (kg/m^2^) and categorized into normal weight (BMI < 25.0 kg/m^2^), overweight (BMI 25–29.9 kg/m^2^) and obesity (BMI ≥ 30 kg/m^2^) [18]. Smoking status was categorized into never, former and current smokers. Alcohol use was categorized into none, moderate and heavy. The limit for heavy alcohol use was set to >16 drinks/week for women, >21 drinks/week for men which correspond with the medium risk levels of daily consumption set by the World Health Organization [19].

Disease status was constructed by taking into account chronic diseases in all pre-retirement waves available. Data on chronic illnesses was based on eligibility for special reimbursement based on the Social Insurance Institution of Finland’s Drug Reimbursement Register (asthma, diabetes, rheumatoid arthritis, coronary heart disease and depression), the Finnish Cancer Registry (cancer) and the questionnaires (osteoarthritis). For the analyses, the number of chronic diseases were modelled as a time-variant variable and participants were categorized as having no disease, one disease and two or more diseases.

### Statistical analyses

Characteristics of the study population before retirement (year −2) for each retirement type are presented as mean values for continuous variables and as proportions for categorical variables. Trajectories of weekly MET hours (continuous outcome) were assessed using linear regression analyses with generalized estimation equations (GEE), and trajectories of physical inactivity (binary outcome) using log-binominal regression analysis with GEE. The GEE models control for the intra-individual correlation between repeated measurements using an exchangeable correlation structure and is not sensitive to measurements missing completely at random [20, 21].

To examine whether weekly average MET hours differed between pre-retirement period (years −10 to −6), during retirement transition (years −2 and +2) and post-retirement period (years +6 to +10), we tested period x time interaction effects separately for the different retirement types. In addition, interaction test of retirement type x period x time was conducted to examine the differences between retirement types. Adjusted mean estimates and their 95 % confidence intervals were calculated to represent an average of 4-year change of weekly total MET hours and change of hours of moderate-level and vigorous physical activity at different periods within retirement type. The analyses were adjusted for retirement age, sex, occupational status, life-style factors and number of chronic conditions before retirement.

We also examined whether retirement age, sex, occupational status and number of chronic conditions were associated with changes in weekly MET hours and physical inactivity during retirement transition and post-retirement by using contrast statements in GEE models. The models were adjusted for retirement age, sex, occupational status, life-style factors and number of chronic conditions before retirement.

Finally, to examine the role of selection (attrition bias), we conducted sensitivity analyses by repeating all analyses among those who had all four physical activity measurements available. The SAS 9.4 Statistical Package was used for all of the analyses (SAS Institute Inc., Cary, NC).

## Results

The average retirement age varied significantly between retirement types being 61.9 (SD 2.0) years among those entering to statutory retirement, 58.9 (SD 2.0) years among part-time retirees and 55.4 (SD 5.8) years among disability retirees. Characteristics of the study population are shown in Table 1.

**Table 1:** Characteristics of the study population before retirement by retirement type

	Statutory retirement		Part-time retirement		Disability retirement		*p*-value
	*n* = 5770		*n* = 1587		*n* = 2131		
Retirement age (mean, SD)	61.92	2.00	58.93	2.02	55.38	5.80	< .0001
	*n*	%	*n*	%	*n*	%	
Retirement age							
< 60	686	11.9	1112	70.1	1721	80.8	
60–64	4239	73.5	460	29.0	410	19.2	
> 64	845	14.6	15	1.0	0	0.0	
Sex							
Men	1160	20.1	343	21.6	350	16.4	< .0001
Women	4610	79.9	1244	78.4	1781	83.6	
Occupational status							< .0001
Upper grade non-manual	2205	38.4	534	33.8	340	16.2	
Lower grade non-manual	1545	26.9	453	28.7	587	27.7	
Manual	1992	34.7	593	37.5	1195	56.3	
Number of chronic diseases							< .0001
0	288	5.0	99	6.2	106	5.0	
1	3060	53.0	806	50.8	647	30.4	
> 1	2422	42.0	682	43.0	1378	64.7	
Smoking status							< .0001
Never	4208	74.56	1065	68.8	1286	60.04	
Former	940	16.65	307	19.83	390	18.81	
Current	496	8.79	176	11.37	397	19.15	
Alcohol use							< .0001
None	909	15.8	255	16.2	443	20.9	
Moderate	4394	76.6	1184	75.1	1528	72.0	
Heavy	437	7.6	138	8.8	152	7.2	
Body Mass Index							< .0001
Normal weight (< 25 kg/m^2^)	2393	43.1	665	43.6	759	36.8	
Overweight (25–29.9 kg/m^2^)	2266	40.8	594	39.0	806	39.1	
Obese (≥ 30 kg/m^2^)	890	16.04	266	17.44	499	24.2	

### Changes in physical activity around retirement

**Fig. 1:**
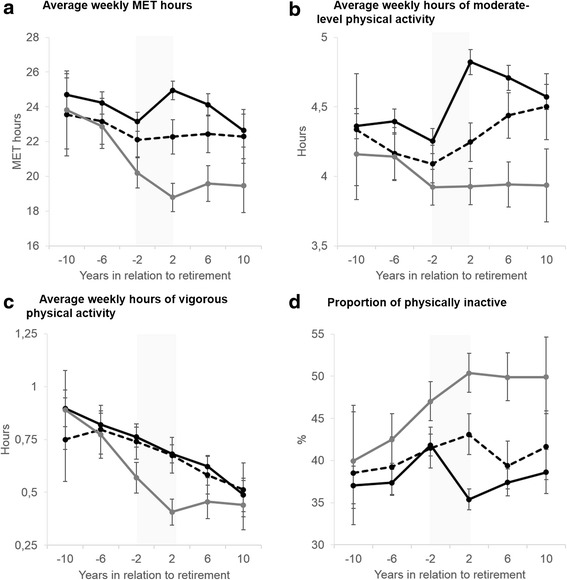
Physical activity trajectories during retirement transition by retirement type. Adjusted for retirement age, sex and occupational status. Legends: Black line: statutory retirement; dashed black line: part-time retirement and gray line: disability retirement. **a** Average weekly MET hours, **b** Average hours of moderate-level physical activity, **c** Average hours of vigorous physical activity, **d** Proportion of physically inactive

Figure 1a shows the trajectories of weekly MET hours before, during and after retirement by retirement type. The slopes for weekly MET hours differed across retirement types and period (retirement type x period x time interaction *p* = 0.001). Table 2 shows changes in total, moderate and vigorous level physical activity among those transitioning from full-time work to statutory retirement. At the measurement before retirement (wave -1) average weekly MET hours were 23.1 (95 % CI 22.6 to 23.7). During the four year retirement transition a statistically significant increase was observed (1.81 MET hour, 95 % CI 1.20 to 2.42). During the post-retirement period (from years +6 to wave +10) statistically significant decline was observed (−1.80 MET hour, 95 % −2.82 to −0.79). In the post-retirement period the average physical activity level among statutory retirees was 0.97 MET hours (95 % CI 0.09 to 1.85) lower than in pre-retirement period.

**Table 2:** Change in total, moderate and vigorous level physical activity and their 95 % CI at different point of retirement transition among those entering to statutory retirement

	Time in relation to retirement									
	Pre-retirement			Retirement transition			Post-retirement			
	Mean change^a^	95 % CI		Mean change^a^	95 % CI		Mean change^a^	95 % CI		Interaction: period x time, *p*-value
Total weekly physical activity (MET hours)	−0.50	−1.54	0.53	1.81	1.20	2.42	−1.81	−2.83	−0.79	< .0001
Moderate-level physical activity (hours/week)	0.03	−0.11	0.18	0.58	0.49	0.67	−0.17	−0.32	−0.01	< .0001
Vigorous physical activity (hours/week)	−0.07	−0.17	0.02	−0.08	−0.14	−0.03	−0.16	−0.25	−0.07	0.29

Notes: Models adjusted for retirement age, sex, occupational status, smoking, alcohol use, BMI and number of chronic diseases before retirement

^a^Change is estimated over four years of time

In addition to total physical activity, trajctories of moderate and vigorous level physical activity were examined (Fig. 1b and c and Table 2). Among those retiring on a statutory basis, weekly hours of moderate-level physical activity increased markedly during the retirement transition (0.58 h, 95 % CI 0.49 to 0.67) followed by a small decline (−0.17 h, 95 % CI −0.32 to −0.01) in post-retirement. However, the activity level in post-retirement period was still 0.32 h per week (95 % CI 0.19–0.45) higher than in pre-retirement period. Vigorous activity, on the other hand, declined with increasing age with no apparent break in the trend around retirement.

Trajectories related to the prevalence of physical inactivity are shown in Fig. 1d. The slope for physical inactivity during pre-retirement, retirement transition and post-retirement was different for the three retirement groups (test of interaction *p* = 0.04). During the retirement transition those retiring on a statutory basis became less inactive (i.e., the prevalence of inactivity decreased from 42 % to 35 %, *p* < .0001), but during the post-retirement there was no statistically significant change in physical inactivity.

Physical activity trajectories (total, moderate, vigorous and inactivity) were also examined separately for men and women retiring on a statutory basis (Additional file 1: Figure S1). Men were physically more active than women, but the trajectories from pre-retirement to retirement transition and to post-retirement were very similar in both genders. This was also supported by sex x time interaction tests, none of which were statistically significant (total *p* = 0.30, moderate *p* = 0.64, vigorous *p* = 0.09 and inactivity *p* = 0.11). Adjustment for chronic disease, smoking, alcohol and BMI did not change the interaction results.

Results related to part-time and disability retirement are shown in online supplement material (Additional file 1: Table S1). Those entering part-time retirement showed no change and those retiring due to disability showed a 1.54 MET hour (95 % CI −2.45 to −0.64) decline over retirement transition.

### Predictors of physical activity change

Table 3 shows the associations of sex, retirement age, occupational status and health status before retirement with change in weekly MET hours during retirement transition and post-retirement period among those entering to statutory retirement. Retirement age > 64 years, higher occupational status and fewer chronic conditions were associated with greater increases in physical activity during retirement transition compared to those retiring younger than 60 years of age, in lower occupational status or with two or more chronic conditions. None of the pre-retirement factors predicted decline in physical activity in the post-retirement period.

**Table 3:** Factors predicting change in weekly physical activity (MET hours) during retirement transition and in post-retirement among those entering to statutory retirement

				Retirement transition			Post-retirement		
	Mean MET hours at pre-retirement	95 % CI		Mean change^a^	95 % CI		Mean change^a^	95 % CI	
Retirement age									
< 60	22.98	21.12	24.84	0.78	−1.10	2.66	−0.40	−2.73	1.93
60–64	20.88	19.92	21.84	1.76	1.05	2.47	−1.43	−2.59	−0.28
> 64	18.98	17.54	20.42	2.91	1.40	4.42	−4.18	−10.94	2.57
Sex									
Men	22.39	20.93	23.85	2.23	0.70	3.76	−1.11	−4.00	1.77
Women	19.30	18.42	20.17	1.71	1.05	2.36	−1.31	−2.38	−0.24
Occupational status									
Upper grade non-manual	20.62	19.43	21.82	2.99	2.06	3.92	−1.55	−3.20	0.10
Lower grade non-manual	21.31	19.98	22.63	0.98	−0.20	2.15	−1.66	−3.62	0.30
Manual	21.18	19.96	22.40	1.12	0.03	2.22	−0.61	−2.36	1.13
Number of chronic diseases before retirement									
0	19.13	16.75	21.52	2.63	−0.06	5.32	0.42	−2.98	3.83
1	21.81	20.83	22.79	1.74	0.90	2.58	−1.02	−2.53	0.50
> 1	20.95	19.88	22.03	1.81	0.88	2.74	−1.83	−3.24	−0.42

Notes: Models adjusted for retirement age, sex, occupational status, smoking, alcohol use, BMI and comorbidity before retirement

^a^Change is estimated over four years of time

Corresponding results for part-time and disability retirement are shown in online supplement material (Additional file 1: Table S2 and S3). Younger retirement age, lower occupational status and co-morbidity predicted greater decline in weekly physical activity during retirement transition compared to higher retirement age, upper occupational status and lack of chronic diseases among disability retirees. None of the pre-retirement factors predicted changes during retirement transition and after retirement among part-time retirees.

Finally, to examine the selection in to the study, we repeated the analyses including participants who had all four physical activity measurements available and the results were replicated suggesting no major selection across retirement groups (Additional file 1: Table S4).

## Discussion

In this longitudinal occupational study from Finland, we observed a transient increase in physical activity (mainly moderate-level activity) during retirement transition among those entering to statutory retirement followed by a post-retirement decline. We also found that this increase was greater among those retiring at older ages, from higher status occupations, and with fewer chronic diseases.

Our study agrees with the few earlier findings showing an increase in moderate-level physical activity during the transition to statutory retirement [6, 8]. However, it also expands previous knowledge by suggesting that the observed increase is temporary, and that the overall physical activity levels tend to decline during the years following retirement. This implies that the free time people get when retiring could be a window of opportunity for activity change, but people may need additional support in order to maintain their increased activity levels during the post-retirement years.

While moving into retirement is considered a significant transition in life, very few physical activity intervention studies have focused on this critical period in life [22]. Our results suggests that two potential time periods could be particularly suitable for interventions. Conducting interventions immediately after retirement could boost the natural increase in physical activity. People might be especially receptive to an offer of activities at this point in life [6] and newly adapted behaviors might extend to the years after retirement. In addition, to counteract the post-retirement activity decline observed in our study, it might be also useful to engage people’s activity behavior a few years after retirement, or alternatively extending the support for active lifestyles for a few years after retirement with semiannual boosters. Clearly further studies are needed to examine the optimal timing and suitable types of interventions that could support recently retired persons to maintain or increase their physical activity at recommended levels and promote active and healthy aging in a cost-effective way.

The results related to physical activity among part-time retirees showed very little changes during pre-retirement, retirement transition and post-retirement. This could be explained by the fact that part-time retirees are a very heterogenous group. Some older workers are shifting to part-time retirement to reduce the burden of work, but others have additional, more personal, motives to continue working only part-time (e.g. spouse is ill and requires care-giving). Participants who entered disability-retirement showed a gradual decrease in their physical activity levels, especially during the transition years to retirement. This was expected, because these people have an illness or injury which prevents them from continuing to work, which is also likely to limit their ability to be physically active. Thereby, the findings related to disability retirement are subject to unobserved selection.

Our findings are generally in line with those earlier found that retirement appears to be beneficial for health including better self-rated health [23], better mental health [24] and less headache [25]. The reasons for these favorable changes may be that people are no longer exposed to physically or mentally stressful working conditions and they are able to spend more time engaged in healthy activities, including physical activity.

A major strength of our study is the repeated measurement of physical activity around an objectively determined retirement transition for all participants which enabled us to estimate trajectories of physical activity and the changes in activity patterns before, during and after retirement. By using MET values, we were also able to disentagle the patterns in moderate and vigorous level of physical activity as well as in inactivity. The results suggest that most of the observed changes are related to moderate-level activity, while vigorous activity tends to decline gradually with age and is not influenced by retirement.

The main limitation of this study is the reliance on self-reported physical activity data which are subject to recall and information bias [26]. Furthermore, wedid not examine non-leisure time activities (i.e. at work) or sedentary time. This calls for further studies that measure total physical activity objectively around retirement. For example, accelerometers provide a feasible and accurate method to measure daily physical activity and sedentary time over multiple days [27–29]. This would enable estimating the changes in total physical activity, including work- and household-related activity which are not often captured with questionnaires but have shown to change during retirement [7]. An additional limitation to this study is that we did not have information on participants’ functional status that may affect participation in physical activity. However, functional limitations are relatively rare among people in their late 50’s and early 60’s; as less than one in ten Finnish adults at age 55 to 64 years report difficulties in walking or stair climbing [30]. Thus, changes in physical activity before and during the retirement transition are less likely to have been affected by changes in functional status, although the decline in physical activity observed post-retirement may partly be due to functional decline since mobility difficulties increase with age. To address this limitation further studies should examine the interplay of physical functioning and physical activity after retirement transition. Finally, the generalizability of the findings may be limited as the cohort consisted of public sector employees of Caucasian origin in a Scandinavian welfare state with a relatively genereous retirement scheme.

## Conclusions

This study suggests that the transition to statutory retirement is associated with a slight yet transient increase in moderate level physical activity among public sector workers, which is not maintained after entering to the post-retirement period. Intervention studies are needed to test whether retirement offers an optimal and opportune moment for interventions to increase physical activity especially among those transitioning to statutory retirement.

### Ethics approval and consent to participate

The FPS study was approved by the Ethics Committee of the Hospital District of Helsinki and Uusimaa. The participants gave their informed consent to take part when responding to the questionnaires.

### Consent for publication

Not applicable.

### Availability of data and materials

Data for research purposes are available upon request.

## Additional file

Additional file 1:
**Table S1.** Change in moderate and vigorous level physical activity (hours/week) and its 95 % CI at different point of retirement transition among those transitioning to part-time and disability retirement. **Table S2.** Factors predicting change in weekly physical activity (MET hours) during retirement transition and in post-retirement among those entering to part-time retirement. **Table S3.** Factors predicting change in weekly physical activity (MET hours) during retirement transition and in post-retirement among those entering to disability retirement. **Table S4.** Change in total, moderate and vigorous level physical activity and their 95 % CI at different point of retirement transition by retirement type. Only participants with four observations are included. **Figure S1.** Physical activity trajectories during retirement transition by sex. Adjusted for retirement age and occupational status a) Average weekly MET hours b) Average hours of moderate-level physical activity c) Average hours of vigorous physical activity d) Proportion of physically inactive. (DOCX 276 kb)
