# Supplementary material for: Changes in physical activity during transition to retirement: a cohort study
Source: Int J Behav Nutr Phys Act. 2016 Apr 16;13:51. doi: 10.1186/s12966-016-0375-9 (PMC4833915; doi:10.1186/s12966-016-0375-9)

**Additional file 1**

**Table S1. Change in moderate and vigorous level physical activity (hours/week) and its 95% CI at different point of retirement transition among those transitioning to part-time and disability retirement.**

|  | Time in relation to retirement | | | | | | | | |  |
| --- | --- | --- | --- | --- | --- | --- | --- | --- | --- | --- |
|  | Pre-retirement | | | Retirement transition | | | Post-retirement | | |  |
|  | Mean change* | 95% CI | | Mean change* | 95% CI | | Mean change* | 95% CI | | Interaction: period x time, p-value |
| Total weekly physical activity (MET hours) |  |  |  |  |  |  |  |  |  |  |
| Part-time retirement | -0.54 | -2.98 | 1.89 | -0.09 | -1.19 | 1.00 | -0.31 | -1.98 | 1.35 | 0.93 |
| Disability retirement | -0.73 | -3.11 | 1.65 | -1.54 | -2.45 | -0.64 | -0.15 | -1.87 | 1.56 | 0.38 |
|  |  |  |  |  |  |  |  |  |  |  |
| Moderate-level physical activity (hours / week) |  |  |  |  |  |  |  |  |  |  |
| Part-time retirement | -0.18 | -0.59 | 0.23 | 0.14 | -0.01 | 0.30 | 0.09 | -0.17 | 0.34 | 0.35 |
| Disability retirement | 0.02 | -0.32 | 0.36 | -0.01 | -0.14 | 0.13 | -0.05 | -0.33 | 0.24 | 0.95 |
|  |  |  |  |  |  |  |  |  |  |  |
| Vigorous physical activity (hours / week) |  |  |  |  |  |  |  |  |  |  |
| Part-time retirement | 0.03 | -0.18 | 0.24 | -0.09 | -0.18 | 0.01 | -0.10 | -0.24 | 0.04 | 0.58 |
| Disability retirement | -0.10 | -0.29 | 0.10 | -0.17 | -0.25 | -0.10 | 0.01 | -0.12 | 0.14 | 0.08 |

Notes: Models adjusted for retirement age, sex, occupational status and smoking, alcohol use, BMI and comorbidity before retirement. *Change is estimated over four years of time.

**Table S2. Factors predicting change in weekly physical activity (MET hours) during retirement transition and in post-retirement among those entering to part-time retirement.**

|  |  | | |  | Retirement transition | | |  | Post-retirement | | |
| --- | --- | --- | --- | --- | --- | --- | --- | --- | --- | --- | --- |
|  | Mean MET hours at pre-retirement | 95% CI | |  | Mean change* | 95% CI | |  | Mean change* | 95% CI | |
| Sex |  |  |  |  |  |  |  |  |  |  |  |
| Men | 18.22 | 15.92 | 20.51 |  | 1.97 | -0.26 | 4.20 |  | -1.53 | -5.11 | 2.06 |
| Women | 19.53 | 17.95 | 21.12 |  | -0.66 | -1.92 | 0.59 |  | 0.61 | -1.30 | 2.52 |
|  |  |  |  |  |  |  |  |  |  |  |  |
| Retirement age |  |  |  |  |  |  |  |  |  |  |  |
| < 60 | 19.94 | 18.28 | 21.59 |  | -0.68 | -1.92 | 0.56 |  | 1.13 | -0.70 | 2.96 |
| 60–64 | 18.53 | 16.33 | 20.74 |  | 1.21 | -1.01 | 3.42 |  | -3.11 | -7.22 | 1.00 |
| > 64 | 20.72 | 10.83 | 30.62 |  | 3.81 | -13.10 | 20.72 |  | Non-est |  |  |
|  |  |  |  |  |  |  |  |  |  |  |  |
| Occupational status |  |  |  |  |  |  |  |  |  |  |  |
| Upper grade non-manual | 19.63 | 15.39 | 23.87 |  | -0.37 | -2.27 | 1.53 |  | 0.99 | -1.92 | 3.90 |
| Lower grade non-manual | 19.14 | 15.04 | 23.24 |  | 0.95 | -0.81 | 2.71 |  | 1.11 | -2.45 | 4.66 |
| Manual | 20.37 | 16.04 | 24.70 |  | -0.66 | -2.61 | 1.30 |  | -1.32 | -3.73 | 1.10 |
|  |  |  |  |  |  |  |  |  |  |  |  |
| Number of chronic diseases before retirement |  |  |  |  |  |  |  |  |  |  |  |
| 0 | 19.97 | 15.08 | 24.86 |  | -3.27 | -8.24 | 1.70 |  | 3.38 | -6.21 | 12.96 |
| 1 | 21.17 | 19.32 | 23.01 |  | 0.16 | -1.38 | 1.69 |  | -1.50 | -3.78 | 0.77 |
| >1 | 19.46 | 17.70 | 21.23 |  | 0.05 | -1.59 | 1.69 |  | 1.72 | -0.79 | 4.23 |

Notes: Models adjusted for retirement age, sex, occupational status, smoking, alcohol use, BMI and comorbidity before retirement. *Change is estimated over four years of time.

**Table S3. Factors predicting change in weekly physical activity (MET hours) during retirement transition and in post-retirement among those entering to disability retirement.**

|  |  | | |  | Retirement transition | | |  | Post-retirement | | |
| --- | --- | --- | --- | --- | --- | --- | --- | --- | --- | --- | --- |
|  | Mean MET hours at pre-retirement | 95% CI | |  | Mean change* | 95% CI | |  | Mean change* | 95% CI | |
| Sex |  |  |  |  |  |  |  |  |  |  |  |
| Men | 18.73 | 16.39 | 21.07 |  | -1.50 | -3.87 | 0.86 |  | -3.31 | -7.54 | 0.92 |
| Women | 18.33 | 17.16 | 19.50 |  | -1.55 | -2.53 | -0.57 |  | -0.10 | -2.01 | 1.80 |
|  |  |  |  |  |  |  |  |  |  |  |  |
| Retirement age |  |  |  |  |  |  |  |  |  |  |  |
| < 60 | 19.01 | 17.60 | 20.42 |  | -2.31 | -3.29 | -1.33 |  | -0.21 | -2.18 | 1.75 |
| 60–64 | 17.96 | 15.76 | 20.16 |  | 1.67 | -0.61 | 3.95 |  | -2.41 | -5.94 | 1.13 |
| > 64 | Non-est |  |  |  | Non-est |  |  |  | Non-est |  |  |
|  |  |  |  |  |  |  |  |  |  |  |  |
| Occupational status |  |  |  |  |  |  |  |  |  |  |  |
| Upper grade non-manual | 17.43 | 15.29 | 19.58 |  | -0.18 | -2.04 | 1.67 |  | 0.02 | -3.28 | 3.32 |
| Lower grade non-manual | 19.83 | 17.58 | 22.09 |  | -2.23 | -4.02 | -0.44 |  | -2.23 | -5.26 | 0.80 |
| Manual | 19.07 | 17.48 | 20.66 |  | -1.60 | -2.84 | -0.35 |  | 0.02 | -2.57 | 2.62 |
|  |  |  |  |  |  |  |  |  |  |  |  |
| Number of chronic diseases before retirement |  |  |  |  |  |  |  |  |  |  |  |
| 0 | 18.65 | 14.28 | 21.68 |  | 1.64 | -2.63 | 5.90 |  | -9.44 | -3.71 | 2.11 |
| 1 | 19.72 | 17.72 | 21.72 |  | -2.61 | -4.39 | -0.83 |  | 1.02 | -2.08 | 4.11 |
| >1 | 17.08 | 15.28 | 18.87 |  | -1.27 | -2.35 | -0.20 |  | -0.63 | -2.84 | 1.59 |

Notes: Models adjusted for retirement age, sex, occupational status, smoking, alcohol use, BMI and comorbidity before retirement. *Change is estimated over four years of time.

**Table S4. Change in total, moderate and vigorous level physical activity and their 95% CI at different point of retirement transition by retirement type. Only participants with four observations are included.**

|  | Time in relation to retirement | | | | | | | | |  |
| --- | --- | --- | --- | --- | --- | --- | --- | --- | --- | --- |
|  | Pre-retirement | | | Retirement transition | | | Post-retirement | | |  |
|  | Mean change* | 95% CI | | Mean change* | 95% CI | | Mean change* | 95% CI | | Interaction: period x time, p-value |
| Total weekly physical activity (MET hours) |  |  |  |  |  |  |  |  |  |  |
| Statutory retirement | -0.52 | -1.58 | 0.55 | 1.96 | 1.26 | 2.67 | -1.44 | -2.48 | -0.39 | <.0001 |
| Part-time retirement | 0.01 | -2.39 | 2.40 | -0.13 | -1.48 | 1.22 | -0.27 | -2.01 | 1.47 | 0.90 |
| Disability retirement | -1.25 | -3.69 | 1.19 | -0.66 | -1.93 | 0.61 | -0.49 | -2.29 | 1.30 | 0.95 |
|  |  |  |  |  |  |  |  |  |  |  |
| Moderate-level physical activity (hours / week) |  |  |  |  |  |  |  |  |  |  |
| Statutory retirement | 0.06 | -0.09 | 0.21 | 0.62 | 0.52 | 0.72 | -0.12 | -0.28 | 0.04 | <.0001 |
| Part-time retirement | -0.12 | -0.55 | 0.32 | 0.15 | -0.05 | 0.34 | 0.09 | -0.17 | 0.35 | 0.36 |
| Disability retirement | -0.01 | -0.37 | 0.36 | 0.09 | -0.11 | 0.28 | -0.06 | -0.35 | 0.24 | 0.75 |
|  |  |  |  |  |  |  |  |  |  |  |
| Vigorous physical activity (hours / week) |  |  |  |  |  |  |  |  |  |  |
| Statutory retirement | -0.08 | -0.18 | 0.02 | -0.09 | -0.15 | -0.03 | -0.15 | -0.24 | -0.06 | 0.31 |
| Part-time retirement | 0.07 | -0.14 | 0.28 | -0.11 | -0.23 | 0.01 | -0.11 | -0.25 | 0.04 | 0.57 |
| Disability retirement | -0.14 | -0.34 | 0.06 | -0.12 | -0.23 | -0.01 | -0.02 | -0.15 | 0.12 | 0.43 |

Notes: Models adjusted for retirement age, sex, occupational status and smoking, alcohol use, BMI and comorbidity before retirement. *Change is estimated over four years of time.

# Figure S1. Physical activity trajectories during retirement transition by sex. Adjusted for retirement age and occupational status. Legends: Black line: men; dashed black line: women.


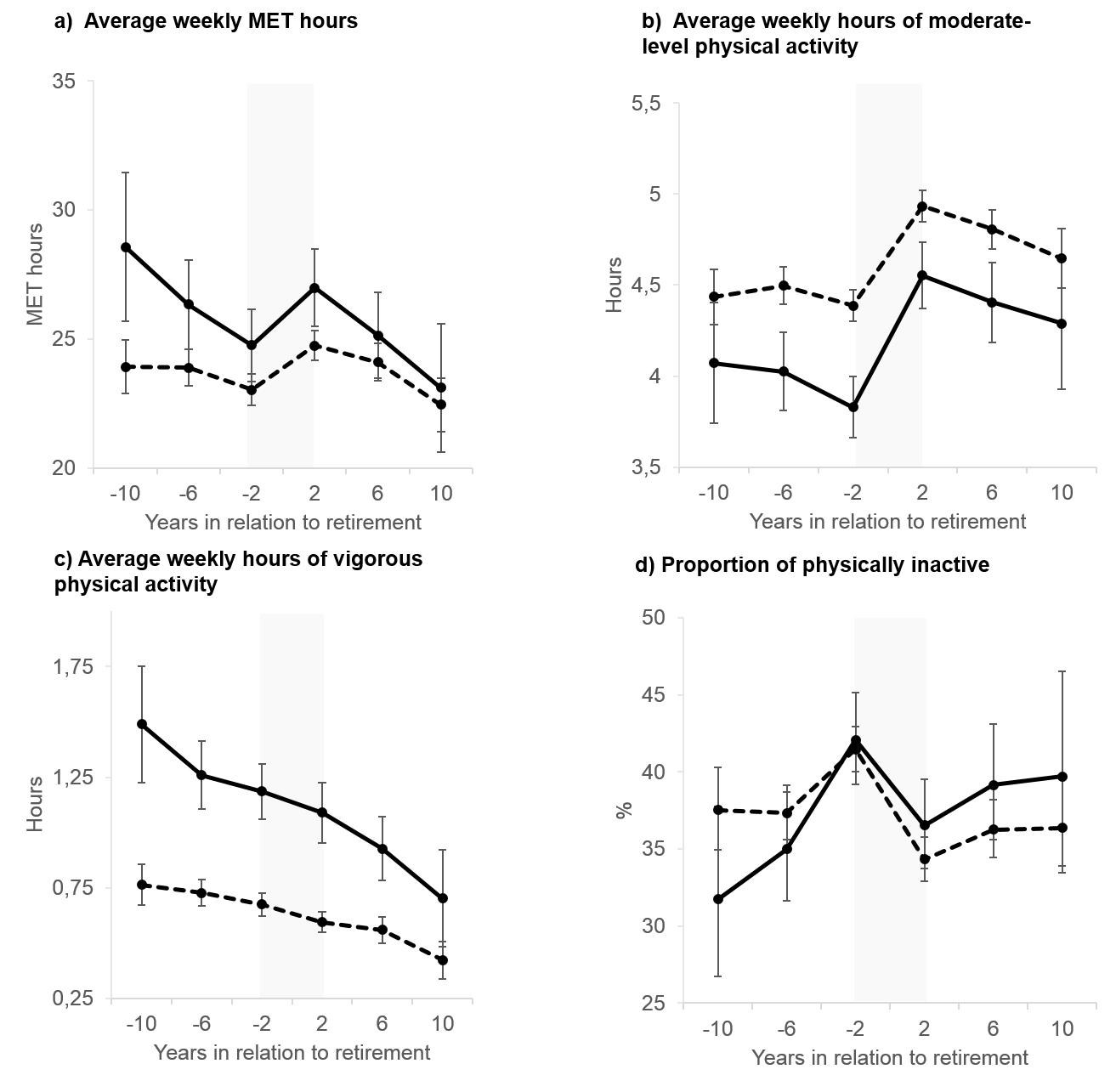

Supplement: Additional file 1: — Table S1. Change in moderate and vigorous level physical activity (hours/week) and its 95 % CI at different point of retirement transition among those transitioning to part-time and disability retirement. Table S2. Factors predicting change in weekly physical activity (MET hours) during retirement transition and in post-retirement among those entering to part-time retirement. Table S3. Factors predicting change in weekly physical activity (MET hours) during retirement transition and in post-retirement among those entering to disability retirement. Table S4. Change in total, moderate and vigorous level physical activity and their 95 % CI at different point of retirement transition by retirement type. Only participants with four observations are included. Figure S1. Physical activity trajectories during retirement transition by sex. Adjusted for retirement age and occupational status a) Average weekly MET hours b) Average hours of moderate-level physical activity c) Average hours of vigorous physical activity d) Proportion of physically inactive. (DOCX 276 kb) [file 12966_2016_375_MOESM1_ESM.docx]
